# Polylactide/Hydroxyapatite Nonwovens Incorporated into Chitosan/Graphene Materials Hydrogels to Form Novel Hierarchical Scaffolds

**DOI:** 10.3390/ijms21072330

**Published:** 2020-03-27

**Authors:** Karolina Kosowska, Patrycja Domalik-Pyzik, Małgorzata Krok-Borkowicz, Jan Chłopek

**Affiliations:** Department of Biomaterials and Composites, Faculty of Materials Science and Ceramics, AGH University of Science and Technology, Al. Mickiewicza 30, 30-059 Krakow, Poland; kosowska@agh.edu.pl (K.K.); krok@agh.edu.pl (M.K.-B.); chlopek@agh.edu.pl (J.C.)

**Keywords:** polylactide, chitosan, polymer composite, electrospinning, hydrogel

## Abstract

In this study, hierarchical, cylindrical scaffolds based on polylactide (PLA) microfibers incorporated into chitosan (CS) hydrogel were prepared for potential use in bone tissue engineering. PLA nonwovens modified with hydroxyapatite particles (HAp) were obtained using the electrospinning method. Then, three-dimensional scaffolds were created by rolling up the nonwovens and immersing them in CS-based solutions with graphene oxide (GO) or reduced graphene oxide (rGO) dispersed in the polymer matrix. Hydrogels were cross-linked using a novel freezing-thawing-gelling method. A broad spectrum of research methods was applied in order to thoroughly characterize both the nanofillers and the composite systems: scanning electron microscopy, X-ray photoelectron spectroscopy, X-ray diffractometry, attenuated total reflection Fourier transform infrared spectroscopy, rheological and mechanical testing, as well as the assessment of chemical stability, bioactivity and cytocompatibility.

## 1. Introduction

Bone can be described as a hierarchical, multi-scale composite consisting of nanocrystalline hydroxyapatite, organic parts (mainly fibers made of collagen type I) and water [1]. The nanocomposite architecture provides great mechanical properties in bone, including high hardness and compressive strength. The extracellular matrix supports the proliferation and differentiation of bone cells [2]. Numerous processes take place in bone tissue, such as the continuous replacement of damaged fragments with new ones, and the transport of nutrients and metabolic waste. However, there is a certain type of damage to the bone that cannot heal by itself, i.e., critical size defect (CSD). In this case, various treatments are used, such as bone grafting, implantation of biomaterials [3], or the application of drugs [4] and growth factors [5].

The tissue engineering approach offers a possibility to regenerate CSD with the use of biodegradable scaffolds with a gradient and hierarchical structure that mimics bone tissue. Following the biomimetic approach, various composite materials are tested for tissue engineering applications. For example, hydrogels based on proteins (collagen [6,7,8]) and polysaccharides (chitosan [9,10,11], alginate [12,13]) are often used as composite matrices. Hydrogel is a network of polymer chains, and can contain a large amount of water [14]. The hydrated structure resembles the natural cell environment—the extracellular matrix. However, hydrogels often show poor mechanical properties that deviate from those of bone tissue. There are many methods to modify the physicochemical properties of hydrogels, such as the incorporation of appropriate nanofillers [15,16,17] or the use of various types of cross-linkers [18]. Recently, graphene family materials (GFM) have gained a lot of attention in tissue engineering [19,20]. In our previous work, we used the unique properties of graphene oxide (GO) to fabricate a chitosan hydrogel with significantly improved mechanical properties [21]. We have developed a method of simultaneous reduction of GO and self-organization of the composite components. Other scientists have also reported the positive effect of GFM on the physical and biological properties of chitosan-based hydrogels [9,16].

The second currently intensively investigated group of materials are fibrous polymeric mats made using the electrospinning method [22]. Electrospinning uses electrical force to draw charged threads of polymer solution from a syringe. The solvent evaporates and thin fibers settle on a grounded collector. The process is relatively cheap, and the diameter and direction of fiber arrangement can be controlled by parameters such as concentration of a polymer solution, voltage, or collector rotation speed. For bone tissue engineering applications, poly(L-lactide) (PLA) [23,24], poly(ε-caprolactone) (PCL) [25,26] and poly(ethylene glycol) (PEG) [27] are most commonly used. However, even when cell tests confirm the biocompatibility of nonwovens, their applicability is limited by their form. They are two-dimensional materials with a dense packing of fibers, which limits cell infiltration. Nonetheless, the important advantages of synthetic polymers when compared to the natural ones, are their favorable mechanical properties. Therefore, they are often the first-choice materials in many biomedical applications [28,29,30,31].

The combination of hydrogels and fibrous mats from electrospinning into three-dimensional structures is a new strategy, currently extensively studied by many research groups [32,33]. Synthetic polymer-based nonwovens act as a reinforcement phase, and thanks to the multilevel design of the nanocomposite, scaffolds mimic the hierarchical structure of bone tissue. Mohabatpour et al. [34] proposed a fiber/hydrogel composite for cartilage tissue engineering. In the study, an electrospun PLA mat was fragmented through aminolysis reaction and incorporated into an alginiate-*graft*-hyaluronate hydrogel and then cross-linked with calcium chloride. The incorporation of fragmented fibers improved the compressive modulus by about 81%. However, this strategy does not increase the porosity of scaffold, which is necessary to improve cells’ infiltration. Jiang et al. [35] fabricated a highly porous composite by the parallel arrangement of PCL nonwovens, immersed in chitosan solution, before cross-linking and freeze-drying. The sandwich was used to guideorient regeneration of periodontal tissue. In another study, an electrospun mat based on poly(hydroxyl butyrate) (PHB) and hydroxyapatite (HAp) was combined with methacrylated gelatin as a matrix. In this case, an advanced method of UV cross-linking was used to form the scaffold [36].

In our work, we proposed three-dimensional, cylindrical scaffolds with a dual pore system. Additional spaces between scaffold walls made of electrospun PLA-HAp nonwoven were designed to improve cell infiltration and the removal of metabolic waste. As matrices, we used chitosan-based hydrogels cross-linked with tannic acid and modified with GO or rGO. Our previous studies have confirmed the positive effect of these modifiers on the mechanical properties of chitosan hydrogels [21]. To form the scaffolds in a desired shape, we used a simple freezing-melting-gelling method. Sodium tripolyphosphate (TPP) in a sodium chloride solution served as a cross-linker.

## 2. Results and Discussion

### 2.1. Graphene Materials

**Figure 1:**
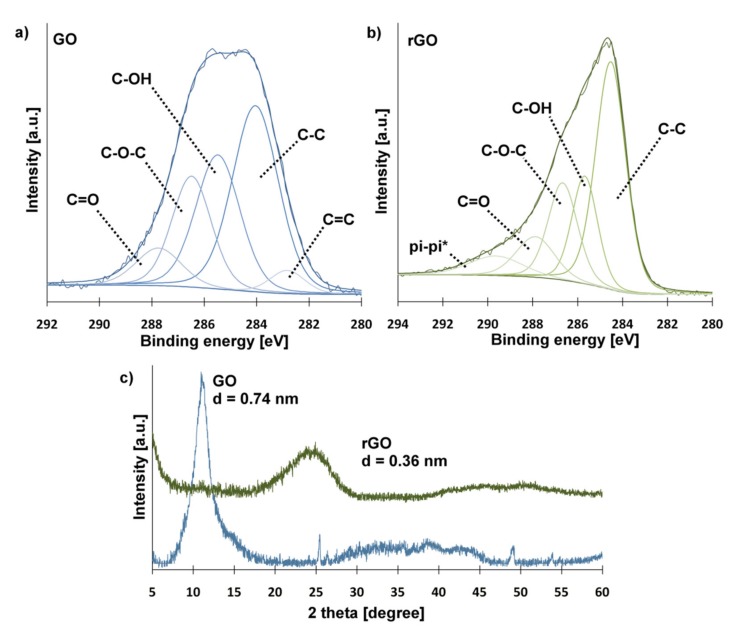
XPS spectra of GO (**a**) and rGO (**b**), XRD spectra of GO and rGO (**c**).

The spectrum of GO, shown in Figure 1a, was fitted into five peaks corresponding to C=O groups (287.8 eV), C-O-C groups (286.5 eV), C-OH groups (285.5 eV), sp^3^ carbon (284.1 eV) and sp^2^ carbon (282.8 eV) [37,38]. The presence of oxygen-containing groups attached to graphene sheets ensures the good dispersibility of GO in aqueous solutions. Furthermore, functional groups can potentially react with amino groups of CS. The C1s spectrum of rGO (Figure 1b) showed additional peak at 289.6 eV corresponding to π-π* interactions between graphene sheets, which suggests the partial reconstruction of the graphene structure. The removal of oxygen-containing groups causes rGO sheets to attach to each other. For this reason, GO and rGO behave differently in solutions and can have a significantly different effect on CS-based hydrogel properties [39]. The degree of GO reduction by L-ascorbic acid was estimated based on the change of C/O atomic ratio derived from XPS analysis. For GO the ratio was 0.95, while for rGO it was 1.30.

The removal of oxygen-containing groups was also confirmed by XRD measurement (Figure 1c). A sharp, single peak at 2θ value 11.13° (d-spacing: 0.74 nm) appeared in the GO pattern. In the case of rGO, the sharp peak disappeared completely, and a new, wide peak appeared at 24.54° (d-spacing: 0.36 nm). The reduction of the d-spacing between carbon sheets confirms the partial removal of oxygen-containing groups [40].

### 2.2. Scaffolds

**Figure 2:**
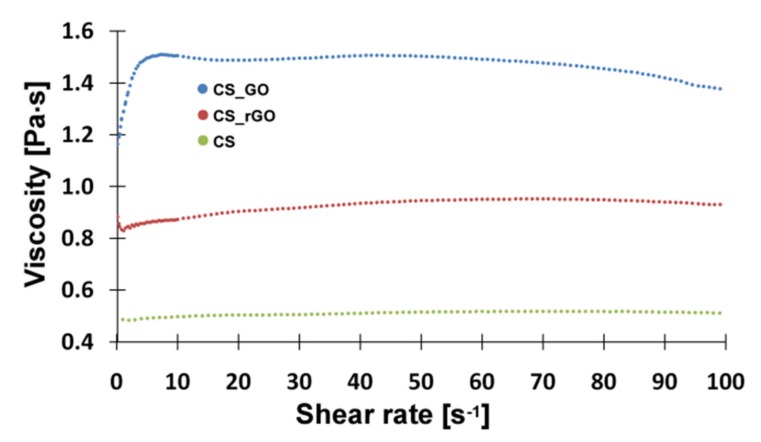
Rheological properties of CS/GO and CS/rGO suspensions.

A rheological analysis was performed to obtain information on the interaction between chitosan chains and the nanofillers. Figure 2 shows that the addition of GO and rGO significantly increased the viscosity of the solution, which suggests an enhanced interaction between the molecules. A higher increase in viscosity was measured for GO, which was probably due to the presence of a higher amount of oxygen-containing groups interacting with the amino groups of CS.

**Figure 3:**
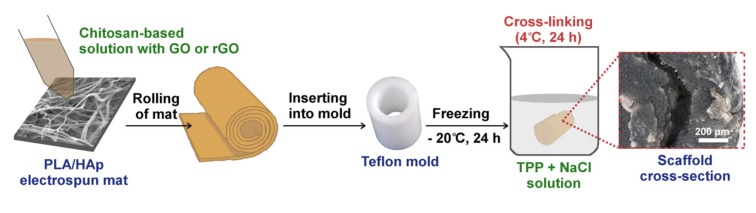
Scheme of three-dimensional scaffold fabrication.

The scaffolds were fabricated using a freezing–thawing–gelling method, as shown in Figure 3. In the first step, the CS-based suspension and rolled-up PLA-HAp nonwoven covered by CS/GO or CS/rGO were frozen in the desired shape. Next, hydrogels were cross-linked in a gelling solution (NaCl + TPP) for 24 h. TPP, tripolyphosphate, is commonly used as a physical cross-linker of chitosan [41,42,43]. However, it is most often added directly to a solution or suspension, which rarely transforms into scaffolds with a desired shape. A low temperature during the gelling process (4 °C) and the high ionic strength of dissolved NaCl prevented the CS solution from thawing too quickly. The protonation of amino groups attached to CS chains under acidic conditions caused electrostatic interaction between NH_3_^+^ ions and Cl^−^ ions from NaCl.

**Figure 4:**
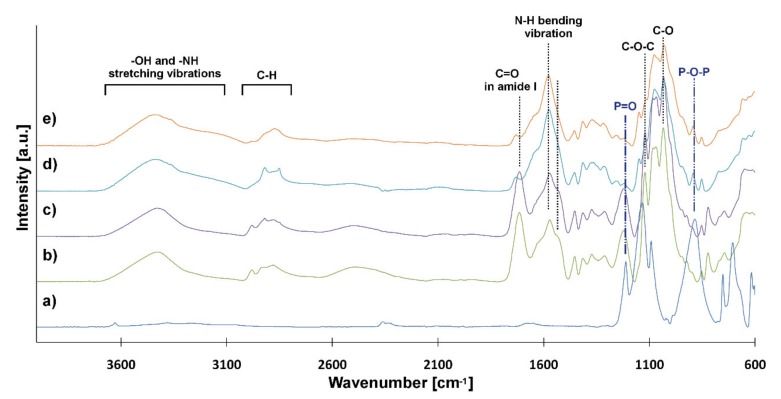
ATR-FTIR spectra: (**a**) TPP, (**b**) CS/GO-TPP, (**c**) CS/rGO-TPP, (**d**) CS/GO, (**e**) CS/rGO.

FTIR-ATR spectra of CS and scaffolds cross-linked with TPP have been analyzed to determine the interaction between CS and TPP (Figure 4). The FTIR spectrum of TPP shows characteristic peaks: at 1212 cm^−1^ (stretching vibration of P=O), at 1130 cm^−1^ (symmetric and anti-symmetric stretching vibration of O-P=O), at 1093 cm^−1^ (symmetric and anti-symmetric stretching vibration of PO_3_) and at 885 cm^−1^ (stretching vibration of P-O-P bridge) [44]. CS was modified by the addition of the nanofillers (GO and rGO) and tannic acid (TAc). TAc is a natural cross-linker containing several –OH groups in the molecule, which can interact with the amino groups of CS [45]. In previous research, we found that TAc addition enhances the mechanical properties of chitosan hydrogels [21]. The spectra of CS/GO and CS/rGO look similar to each other and show characteristic peaks: the overlapped broad peak above 3000 cm^−1^ (–OH and –NH stretching vibrations), at 2885 cm^−1^(C–H stretching vibration), at 1714 cm^−1^ (C=O stretching vibration in amide I -NHCO-) and the broad peak at 1567.6 cm^−1^ (bending vibration of N–H) [44]. The deformation of the last peak can be related to the interaction between CS and TAc and the ionization of amino groups (–NH_2_) under acidic conditions. Another characteristic peak of CS appeared at 1124 cm^−1^ and 1030 cm^−1^, which corresponded to asymmetric stretching vibration in C-O-C bridge and stretching in C-O group, respectively [45]. The spectra of hydrogels cross-linked by TPP revealed some changes. Additional broad peaks can be seen at 1532 cm^−1^. Furthermore, peaks characteristic for TPP were observed at 1211-885 cm^−1^ [41,44]. These confirmed the gelling of CS by TPP as an ionic cross-linking agent. The reaction took place between protonated –NH_3_^+^ groups from CS and P-O^-^ from TPP.

**Figure 5:**
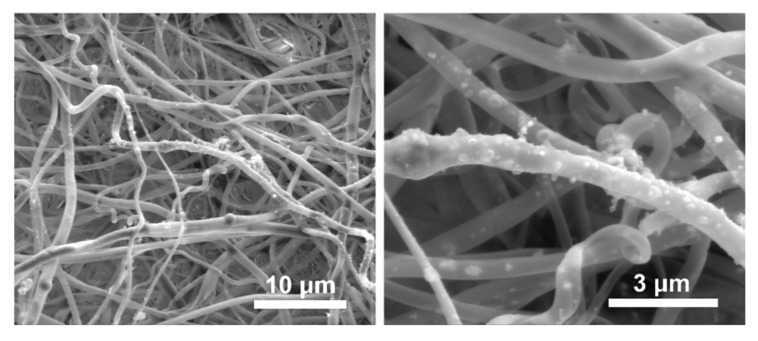
Representative scanning electron microscopy images of PLA-HAp nonwoven.

The design of scaffold has a key impact on the ability of the scaffold to support processes during tissue regeneration, such as cell infiltration, nutrient/waste transport and the formation of new tissue. The aim of our research was to fabricate three-dimensional, cylindrical scaffolds. In the first step, electrospinning was used to create PLA nonwoven modified with bioactive particles in the form of spherical hydroxyapatite (HAp). The synthetic polymer, PLA, was chosen because of the favorable mechanical properties and longer degradation time compared to CS, which should help maintain the desired shape of a scaffold during tissue regeneration. The parameters of ES process were selected to obtain a nonwoven with randomly oriented fibers, as shown in Figure 5. The PLA-HAp fibers were found to be bead-free, with a narrow fiber size distribution. The diameter of the fibers was in the range of 215 nm to 1.34 μm, and the average fiber diameter was 0.70 μm ± 0.23 μm (ImageJ, 100 fibers measured).

**Figure 6:**
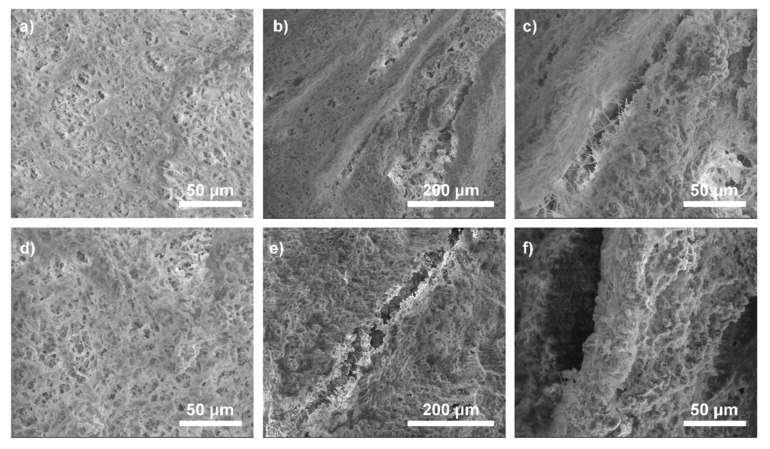
SEM images of CS-based composites and cross-section of the scaffolds with the nonwovens, after freeze-drying: CS/GO (**a**), gaps between walls in CS/GO/PLA-HAp (**b**,**c**), CS/rGO (**d**), gaps between walls in CS/rGO/PLA-HAp (**e**,**f**).

The purpose of pores is to allow the transport of nutrients, the infiltration of cells into scaffolds and the overgrowth of blood vessels. PLA-HAp nonwoven had a network of open pores, but their diameter was mostly around few µm. To improve the porosity, nonwoven was rolled up and covered by CS/GO or CS/rGO solution. After cross-linking, the three-dimensional scaffold with additional porosity was obtained. Figure 6 presents the cross-section architecture of the composite scaffolds. Regardless of the nanofillers used, the scaffold microstructure was similar. It could be seen that additional porosity was obtained between the walls made of the nonwoven. The distance between walls was about 50–200 μm. The diameters of the pores were expected to increase with the CS-based hydrogels degradation.

**Figure 7:**
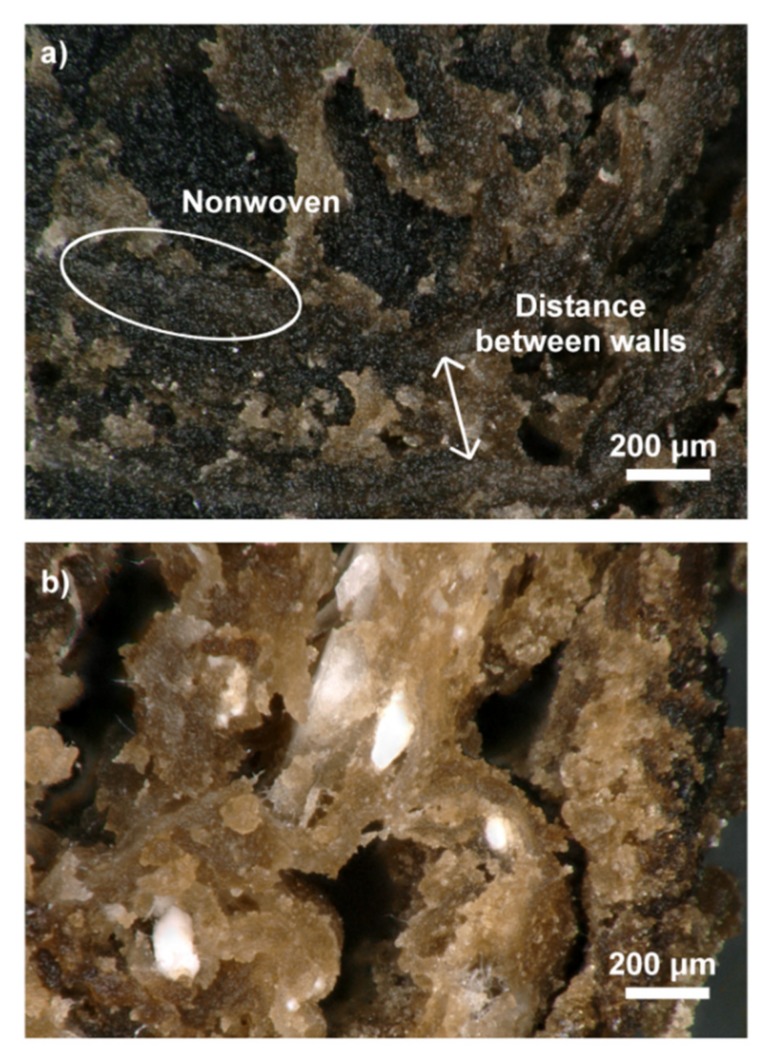
Optical microscope images of cross-section of scaffolds after 3 weeks of degradation in PBS medium: (**a**) CS/rGO/PLA-HAp, (**b**) CS/GO/PLA-HAp.

The in vitro degradation of the hydrogels and the scaffolds in PBS medium was evaluated. After three weeks of incubation, the cross-sections of the samples were investigated using an optical microscope (Figure 7). The results of the study were as anticipated—the CS-based hydrogel used to cover and penetrate wall made with the PLA-HAp nonwoven started to degrade, and as a consequence the pores became bigger.

**Figure 8:**
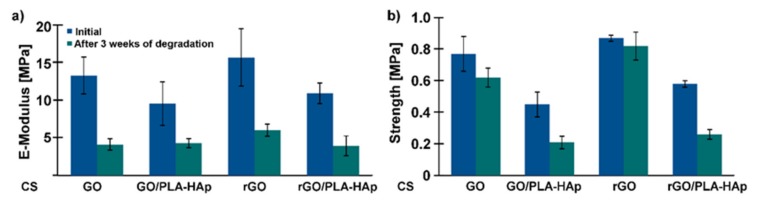
Young’s modulus (**a**) and compression strength (**b**) of the CS-based hydrogels and the scaffolds before and after three weeks of incubation in PBS medium, at 37 °C.

The mechanical properties of the scaffolds were also tested (compression test). In both cases, Young’s modulus was higher for the hydrogels alone than for the hierarchical scaffolds, as shown in Figure 8a. This is related to the lack of porosity. Better mechanical properties of the samples with rGO are believed to be related to more-dense microstructures (Figure 7a). The rGO sheets tend to arrange parallel to each other due to the π-π* interaction with polymer chains interlocked between sheets thanks to the interfacial adhesion of CS and rGO. Furthermore, CS/rGOsuspension had a lower viscosity compared to CS/GO, which could result in the better infiltration of PLA-HAp nonwoven. However, a percentage decrease in Young’s modulus was smaller for the samples with the incorporated nonwovens.

**Figure 9:**
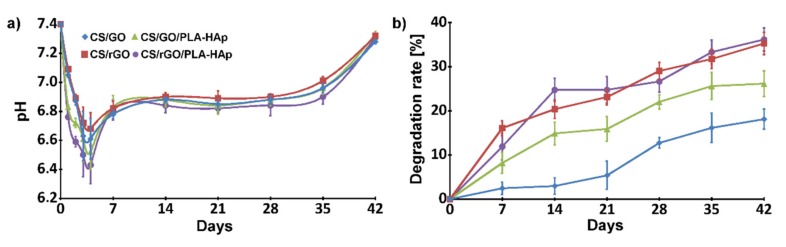
Degradation behavior: pH values of media (**a**) and weight loss of the samples (**b**) as a function of incubation time in PBS.

The degradation of the aliphatic polyester, which is PLA, is much slower than for polysaccharide like CS, and can take a few years in PBS medium. Decrease in PBS pH resulted from the release of LAc from the samples. Due to the presence of pores, the hydrogel surface in direct contact with the medium was larger for scaffolds with nonwovens, thus pH changes were slightly higher (Figure 9a). The degradation rate was also analyzed by measuring the weight of dried samples every week (Figure 9b). GO and rGO had different effects on the stability of the CS-based hydrogels, which was probably related to the presence of oxygen-containing groups attached to GO surface, which interact with amino groups of CS.

**Figure 10:**
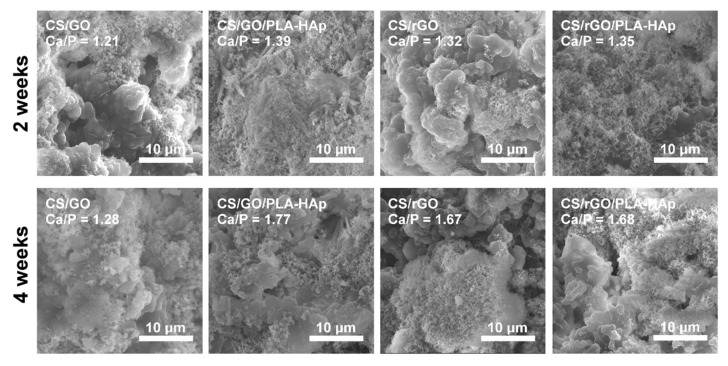
Representative SEM images of the samples surface after 2- and 4-weeks incubation in simulated body fluid, at 37 °C with Ca/P ratios calculated from EDX analyses.

The ability to promote formation of calcium phosphate layer in SBF was investigated in an in vitro bioactivity assay. This phenomenon is particularly important in cases of materials for bone applications, as—to some extent—it can be an indicator of future bone-material bonding ability. As was shown in Figure 10, crystals were formed on all of the samples; however, their morphology and Ca/P ratios differed. After two weeks of incubation, single calcium phosphate clusters in the form of spherical particles were observed on the surface of all samples. The surface area covered with the bone-like apatite layer increased with incubation time. At first, all of the layers had lower Ca/P ratio (in the range of 1.21–1.39) as compared to the typical bone HAp (i.e., Ca/P around 1.67), with the lowest for the CS/GO sample. After another 2 weeks, Ca/P ratio increased significantly for all the tested samples, except for the CS/GO hydrogel.

**Figure 11:**
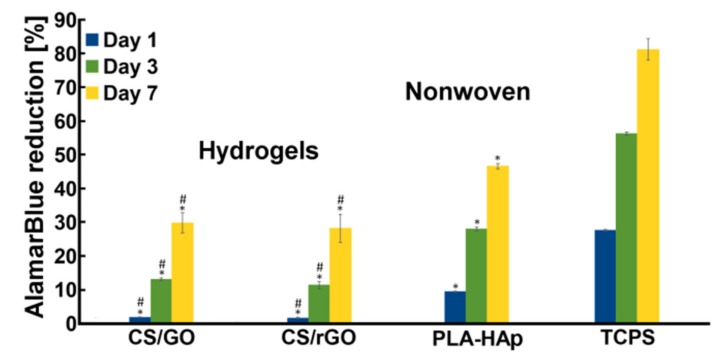
Cell metabolic activity measured by AlamarBlue^®^test—after 24 h, 3 and 7 days. Results: mean ± s.e.m., *n* = 6, statistical significance: at *p* < 0.05 as compared to *TCPS, # nonwoven PLA/HAp.

Preliminary in vitro tests showed that cells adhered to and proliferated on all materials (Figure 11). There were no statistically significant differences in the hydrogels group. Only the tendency that on CS/GO, cells proliferated better than on CS/rGO was observed. Interestingly, the PLA-HAp nonwoven supported cell proliferation to a greater extent than the hydrogel samples.

## 3. Materials and Methods

### 3.1. Materials

High molecular weight (M = 600,000–800,000 g/mol) chitosan (CS) with deacetylation degree >90% and sodium tripolyphosphate (TPP) were purchased from Acros-Organics (Morris Plains, NJ, USA). Lactic acid (LAc, 88%), N,N-dimethylformamide (DMF), dichloromethane (DCM), tannic acid (TAc), NaOH, NaCl and reagents needed for the phosphate buffered saline (PBS) were obtained from Avantor Performance Materials Poland S.A, Poland. Poly(lactic acid) (PLA, IngeoTMBiopolymer 3251D) with molecular weight M = 70,000–120,000 g/mol was bought from NatureWorks LLC (Minnetonka, MN, USA). Graphene oxide (GO) and reduced graphene oxide (rGO)pastes were received from the Institute of Electronic Materials Technology (ITME) (Warsaw, Poland).

### 3.2. Preparation of PLA-HAp Solution

PLA solution for the electrospinning process (ES) with a concentration of 13% (*w*/*v*) was prepared by dissolving polymer in a binary-solvent system of DCM and DMF (2.5:1 *v*/*v*). The solution was stirred for 24 h using magnetic stirrer at about 50 °C. Next, HAp powder was added, and the solution was sonicated for 1 h and then stirred for another 24 h. The concentration of HAp was 6% wt., beyond that content the solution retained its rheological properties.

### 3.3. Electrospinning Process

PLA-HAp nonwoven was formulated through electrospinning process (ES) using apparatus constructed at the Department of Biomaterials and Composites, AGH, Poland. The solution was sonicated for 10 min before injection into 10 mL syringe. A high voltage power supply (0 – 25 kV) was used to generate an electric field between a needle tip and a cylindrical, rotating collector (width: 5 cm) covered with an aluminum foil. Detailed process parameters are summarized in Table 1.

### 3.4. Preparation of Chitosan Hydrogels Modified with GO and rGO

Chitosan solution was prepared by dissolving 2.5 g of CS powder in 5% LAc. The stable suspension of GO was prepared by sonicating 0.644 g paste in 20 mL of deionized water for 2 h. Next, dispersion of GO was added to the CS solution (0.5% of GO to CS weight). After stirring for 24 h, TAc (cross-linker, 10% to CS weight) was added and the whole system was kept stirring for another 24 h. Solutions modified with rGO were prepared in the same way. In order to prepare three-dimensional, cylindrical scaffold, PLA-HAp nonwoven was cut into strips (1.5 cm × 5 cm), then covered by CS/GO or CS/rGO suspension using metal spatula. The needle was used to roll up the nonwoven. Spiral tube was inserted into a cylindrical Teflon mold (width: 5 mm) and frozen at −20 °C for 24 h. The frozen sample was immersed in a gelling solution (0.5% TPP + 5% NaCl in water) at 4 °C for 24 h. Finally, samples were washed with 0.5 M NaOH (0.5 h) and then with deionized water to neutralize LAc and remove impurities. Hydrogels (CS/GO and CS/rGO) were prepared in the same way by injecting the solution into the mold. Finally, two types of cylindrical PLA-HAp nonwoven-reinforced scaffolds (CS/GO/PLA-HAp and CS/rGO/PLA-HAp) and two types of hydrogel scaffolds (CS/GO and CS/rGO) were fabricated. All samples were stored for 24 h before testing.

### 3.5. Characterization of the GO and rGO

X-ray photoelectron spectroscopy (XPS) characterization of graphene materials (GO and rGO) was carried out using spectrometer (Vacuum Systems Workshop Ltd, Crowborough, East Sussex, United Kingdom.) with Mg anode (1253.6 eV Kα radiation; 200 W X-ray excitation source; 3 × 10^−8^ mbar vacuum; 15° electron takeoff angle) in the constant analyzer energy mode (22 eV pass energy). The spectra were fitted with Gaussian–Lorentzian peaks with XPSPEAK 4.1 software (Prof. Raymund W.M. Kwok, The Chinese University of Hong Kong). The X-ray diffraction analysis of the crystal structure was achieved using X’Pert Pro diffractometer with Cu Kα X-ray sources (λ = 1.5406 Å). D-spacing of graphene-based materials was calculated from Bragg equation.

### 3.6. Characterization of the Polymer Solutions

Dynamic rheological measurements were performed using rheometer (MCR 302, Anton Paar) with stainless steel parallel plates (diameter: 20 mm), at controlled, constant temperature 25 °C. The viscosity of solutions was measured in the frequency range between 0.1 and 100 Hz. The test was carried out to evaluate an influence of graphene materials additives on rheological properties of the CS solution.

### 3.7. Characterization of the Scaffolds

The morphology of the scaffolds and PLA-HAp nonwoven was studied using a scanning electron microscope (SEM, Nova NanoSEM 200) with an accelerating voltage of 18 kV. Images of scaffolds were also taken under a digital microscope (VHX-900F, Keyence). The chemical compositions of materials were analyzed using Bruker Tensor 27 spectrometer in ATR mode with diamond crystal. All spectra were taken in the range 4000–600 cm^−1^ (64 scans), at resolution 4 cm^−1^.

### 3.8. Mechanical Properties of the Scaffolds

The mechanical properties of the samples were measured using a universal mechanical tester (Zwick 1435). The compression test was performed with a speed of 1 mm/min. Average values of Young’s Modulus (E) and compressive strength were calculated from at least three independent measurements for each material.

### 3.9. Chemical Stability

The in vitro degradation of the scaffolds was carried out in PBS solution at 37 °C. Samples were immersed in 20 mL of PBS and stored in an incubator for 6 weeks. Once a week, the pH of the medium and the weight of the sample were measured, and PBS was replaced with a fresh one. The weight loss was quantified as the change in the dried sample weight over time:

$$Weight\;loss=\frac{\left(W_{0}-W_{t}\right)}{W_{0}}100\%$$

where W_0_ is the initial weight of the sample and W_t_ is the weight after degradation time.

### 3.10. In Vitro Bioactivity Assay

In the in vitro bioactivity assay, the formation of a calcium phosphate or apatite-like layer on the surface of the samples incubated in simulated body fluid (SBF) was examined. SBF solution was prepared following Bohner’s improved protocol [46]. The samples were immersed in SBF solution (samples surface area to SBF volume was set at 10:1) and kept at 37 °C for 2 and 4 weeks; the solution was replaced weekly. After 2 and 4 weeks, the samples were removed from SBF, rinsed with distilled water and dried at room temperature for SEM/EDX analyses.

### 3.11. Cytocompatibility

The CS/GO and CS/rGO hydrogels were cut into 3-mm-thick slices. Next, samples were incubated in 0.5 NaOH solution for 4 h. NaOH was removed and samples were washed with deionized water three times, for 10 min. After drying at 37 °C for 1 h, hydrogels were sterilized by incubation in 70% ethanol at room temperature for 30 min, and again the samples were dried at 37 °C for 1 h. The electrospun PLA-HAp nonwoven was peeled off from the aluminum foil, incubated in 70% ethanol at room temperature for 30 min and dried overnight at 37 °C. The cell culture was carried out using osteoblasts-like cell MG-63 (European Collection of Cell Cultures, Salisbury, UK) under standard condition, i.e., 37 °C, 5.0% CO_2_, in MEM Eagle medium (PAN BIOTECH, Germany) supplemented by 10% fetal bovine serum (FBS), 1% antibiotics (penicillin/streptomycin), 0,1% amino acid and sodium pyruvate (all from PAA Laboratories Gmbh, Pasching, Oberosterreich, Austria). Cells were seeded at concentration of 15,000 cell/hydrogel and 10,000 cell/nonwoven. Cell number was dependent on the relative surface area of the samples. The incubation time was 24 h, 3 and 7 days. At each time point, cell metabolic activity was checked by AlamarBlue^®^ test (Sigma-Aldrich, Munich, Germany). The samples were tested in triplicate, as a control, TCPS (tissue culture polystyrene) was used. The results were presented as mean ± s.e.m. (standard error of the mean), statistical analysis was carried out by one-way-ANOVA with post-hoc LSD Fisher test (significance level 0.05).

## 4. Conclusions

The three-dimensional, cylindrical scaffolds were successfully obtained by incorporating electrospun, biodegradable PLA-HAp microfibers into the chitosan-based hydrogels. A novel freezing–thawing–gelling method was used to fabricate the cylindrical scaffold with a dual system of porosity. Additional micropores were formed by rolling up the porous nonwoven. Scaffold architecture was designed to improve cells’ penetration and metabolic waste removal. It was also considered that the type of nanofillers used to modify the CS hydrogel had a significant effect on the physicochemical properties and stability of the chitosan matrix. A preliminary, in vitro biological test confirmed the cytocompatibility of the materials. However, further cell studies are necessary.

## Figures and Tables

**Table 1:** Electrospinning parameters.

Voltage (Kv)	Temperature (°C)	Humidity (%)	Collector Rotation (rpm)	Needle-Collector Distance (cm)	Needle Diameter (mm)	Process Time (h)
25	50	10	200	4	0.70	2.5
